# Human Heat shock protein 40 (Hsp40/DnaJB1) promotes influenza A virus replication by assisting nuclear import of viral ribonucleoproteins

**DOI:** 10.1038/srep19063

**Published:** 2016-01-11

**Authors:** Jyoti Batra, Shashank Tripathi, Amrita Kumar, Jacqueline M. Katz, Nancy J. Cox, Renu B. Lal, Suryaprakash Sambhara, Sunil K. Lal

**Affiliations:** 1 School of Science, Monash University, Subang Jaya, Selangor, 47500, Malaysia; 2 Virology Group, International Centre for Genetic Engineering & Biotechnology, New Delhi, 110067, India; 3 Influenza Division, National Center for Immunization and Respiratory Diseases, Centers for Disease Control and Prevention, Atlanta GA, 30333, USA

## Abstract

A unique feature of influenza A virus (IAV) life cycle is replication of the viral genome in the host cell nucleus. The nuclear import of IAV genome is an indispensable step in establishing virus infection. IAV nucleoprotein (NP) is known to mediate the nuclear import of viral genome via its nuclear localization signals. Here, we demonstrate that cellular heat shock protein 40 (Hsp40/DnaJB1) facilitates the nuclear import of incoming IAV viral ribonucleoproteins (vRNPs) and is important for efficient IAV replication. Hsp40 was found to interact with NP component of IAV RNPs during early stages of infection. This interaction is mediated by the J domain of Hsp40 and N-terminal region of NP. Drug or RNAi mediated inhibition of Hsp40 resulted in reduced nuclear import of IAV RNPs, diminished viral polymerase function and attenuates overall viral replication. Hsp40 was also found to be required for efficient association between NP and importin alpha, which is crucial for IAV RNP nuclear translocation. These studies demonstrate an important role for cellular chaperone Hsp40/DnaJB1 in influenza A virus life cycle by assisting nuclear trafficking of viral ribonucleoproteins.

The compact genome of viruses restricts their ability to encode all the proteins required for their efficient replication. In order to circumvent this limitation, viruses depend on the host machinery and often utilize cellular factors to complete vital steps of their life cycle. Cellular chaperones are one of the most commonly targeted classes of host proteins which are subverted by viruses^1^. These ubiquitously expressed proteins include a diverse set of heat shock proteins which play important roles in multiple cellular processes such as protein translation, folding, degradation, intracellular trafficking and stress response^2^,^3^,^4^,^5^. Many viruses co-opt cellular chaperones to assist in viral entry, viral protein synthesis, folding and localization, to regulate viral replication and to interfere with host antiviral responses^6^,^7^,^8^,^9^,^10^. Previous studies have indicated that chaperones can have both positive and negative effects on virus replication^11^,^12^,^13^.

Influenza A viruses are enveloped viruses with negative-sense, single-stranded genome comprised of eight RNA segments. Within virus particle, each viral RNA (vRNA) is covered by multiple copies of nucleoprotein and a single copy of the polymerase heterotrimer (PA, PB1, PB2), thereby constituting a viral ribonucleoprotein (vRNP) complex^14^,^15^,^16^,^17^. IAV NP plays a crucial role in the viral life cycle by interacting with various cellular factors and modulating different signaling pathways. One key function of NP is nuclear trafficking of vRNPs by interacting with importins through its nuclear localization signals^18^,^19^,^20^,^21^,^22^,^23^,^24^,^25^. Also, it has been reported that nuclear export of vRNP is mediated by NEP through its interaction with cellular nucleoporins^26^. Viral protein M1 and NP are known to assist this process via interaction with NEP and cellular CRM1 respectively^27^,^28^.

Hsp40 is a cellular, molecular chaperone that belongs to the heat shock protein family. It is a ubiquitously expressed protein consisting of a highly conserved J domain on N-terminus and substrate recognition domain on C-terminus^29^. Hsp40 has been reported to facilitate nuclear transport of the HIV type 2 Vpx-mediated pre-integration complex^30^. Also, it is important for Nef-mediated enhancement of HIV-1 gene expression and replication^9^. Further, it has been shown to suppress hepatitis B virus replication through destabilization of the viral core and the X protein^11^. In the case of influenza virus, Hsp90 and Hsp70 have been shown to interact with polymerase subunits and therefore have been suggested to be involved in assembly and nuclear transport of viral polymerase subunits, possibly by acting as a molecular chaperone for the viral polymerase complex^31^,^32^. Although few cellular factors involved in nuclear import of influenza viral polymerase complex have been well characterized but many remain to be defined.

Previously, we have shown that Hsp40 interacts with IAV nucleoprotein and this interaction is employed to mitigate PKR mediated antiviral host response^10^. Here we examined the other possible physiological implications of this interaction. In this study, we demonstrated that Hsp40 interacts with NP during early stages of the virus life cycle and facilitates the nuclear translocation of the vRNP complex. The interaction is mediated via the N-terminal domain of NP and J domain of Hsp40. Down-regulation of Hsp40 using chemical inhibitor or Hsp40/DnaJB1 specific siRNA resulted in reduced nuclear accumulation of NP leading to significant reduction in both virus transcription and replication. The effect of Hsp40 inhibition on IAV replication was found to be valid across various IAV strains and in different cell lines. Conversely, an increase in virus replication was observed upon over-expression of Hsp40/DnaJB1. Interestingly, Hsp40 was also found to facilitate the interaction between NP and importin alpha. These findings suggest an important role of cellular chaperone Hsp40/DnaJB1 in the influenza virus replication and establish Hsp40 as a promising antiviral target.

## Results

### Hsp40 associates with incoming influenza A virus vRNPs

**Figure 1:**
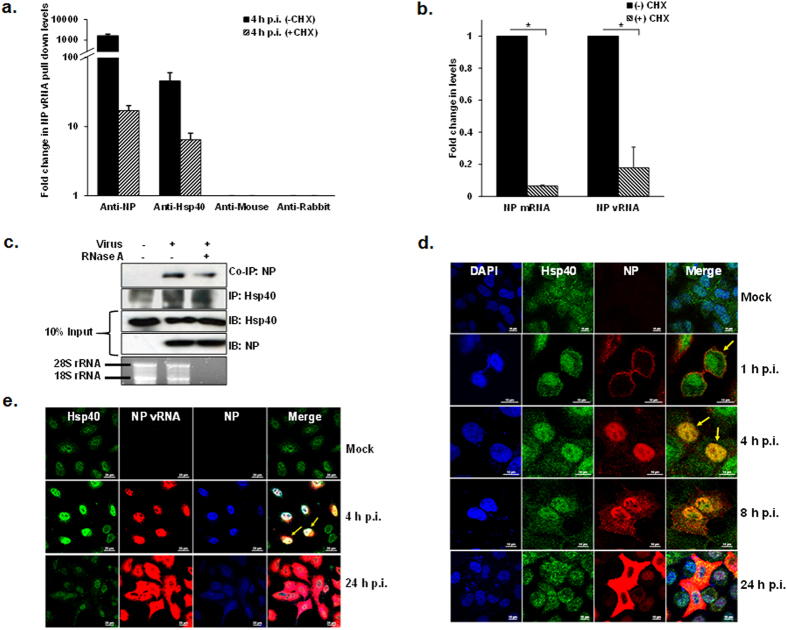
Hsp40 interacts with incoming viral ribonucleoprotein complexes in IAV infected cells. (**a**) Hsp40 interacts with NP component of vRNPs. A549 cells were infected with PR8 virus at MOI = 10 in the presence of cycloheximide and cells were harvested 4 h post-infection. Cell extracts were immunoprecipitated using anti-NP, anti-Hsp40, control IgG mouse and rabbit antibodies. RIP was analyzed with quantitative RT-PCR using NP vRNA specific primers. The fold change in NP vRNA pull down levels was calculated with reference to control antibodies IgG mouse and rabbit. (**b**) Cycloheximide (CHX) treatment significantly reduced NP mRNA and vRNA levels in infected cells. The input sample used to set up RNA immunoprecipitation was tested for NP levels in cycloheximide treated and untreated cells by qRT-PCR. The fold change in NP mRNA and vRNA levels was calculated with reference to CHX untreated cells. (**c**) RNase A treatment partially affects the interaction between NP and Hsp40. A549 cells were infected with virus at MOI = 1 and cells were harvested 24 h post-infection. Cell lysate was treated with RNase (100μg/ml) at 4 °C for 45 min followed by immunoprecipitation using anti-Hsp40 antibody. RNA was extracted from remaining cell lysate and was analyzed on formaldehyde agarose gel. (**d**) NP co-localizes with Hsp40 during early stages of infection. A549 cells were mock infected and infected with PR8 virus at MOI = 5 and were fixed at different time intervals. NP and Hsp40 were stained using anti-NP monoclonal antibody and anti-Hsp40 primary antibody, followed by Alexa 594 and Alexa 488 conjugated secondary antibodies respectively. Arrows indicate the co-localization of NP and Hsp40. (**e**) Hsp40 co-localizes with NP and vRNP. A549 cells were mock infected and infected with PR8 virus at MOI = 5 for 4 h and 24 h. NP and Hsp40 were stained using anti-NP antibody and anti-Hsp40 primary antibody followed by Alexa 350 and Alexa 488 conjugated secondary antibodies respectively. NP vRNAs were detected by positive sense RNA probes using FISH. Arrows indicate the co-localization of IAV NP vRNA, NP and Hsp40. Data show mean ± S.D. from one representative experiment (n = 3) of at least three independent experiments.

Upon IAV entry in to the cells, transport of incoming vRNPs across the cytoplasm to the nucleus is a critical requirement to establish infection. It’s known that IAV proteins may recruit host factors to facilitate this process^22^,^23^,^24^,^25^,^31^,^32^,^33^,^34^,^35^,^36^,^37^. In an earlier report we had shown that IAV NP interacts with cellular Hsp40 in infected cells, which coincides with change in Hsp40 cellular localization from diffused cytoplasmic to primarily nuclear^10^; however it was not known whether Hsp40 interacts with free form or viral ribonucleic acid bound form of NP. To address this question, we performed a RNA immunoprecipitation (IP) assay from cells infected with IAV for 4 hours, using antibodies against IAV NP, Hsp40 and control IgG. To differentiate between incoming NP and de novo translated NP, we treated the cells with translational inhibitor cycloheximide (CHX) to prevent translation of new NP. qRT-PCR analysis revealed the presence of comparable levels of NP vRNA in RNA-protein complexes pulled down with anti-Hsp40 and anti-NP antibodies, from CHX treated cells (Fig. 1a). This indicated that Hsp40 associates with parental vRNPs through nucleoprotein during IAV infection. In CHX untreated cells Hsp40 still immunoprecipitated NP vRNAs, however in much less quantity as compared to NP (Fig. 1a). This may be due to contribution of newly synthesized NP in the IP of vRNPs in absence of CHX. The NP mRNA and vRNA levels were analyzed in cell lysates used to set up IP and were found to be drastically reduced in CHX treated cells as compared to untreated cell lysate (Fig. 1b). We also investigated the role of vRNA in the interaction between NP and Hsp40. The influenza vRNA is RNase-sensitive and NP does not protect vRNA from digestion^38^,^39^,^40^. We treated the IAV infected cell lysates with RNase A to digest any ssRNA present in the preparation. RNA degradation was validated by formaldehyde-agarose gel electrophoresis, which confirmed that both 28S and 16S rRNA species were degraded in the RNase A treated sample (Fig. 1c). Immunoprecipitation was performed using anti-Hsp40 antibody from RNase A treated and untreated cell lysates. We observed that Hsp40-NP interaction was still present after RNase A treatment, however amount of NP co-purified was slightly reduced (Fig. 1c). It indicated that viral RNA may contribute to the NP-Hsp40 interaction quantitatively, probably by facilitating NP oligomerization on the vRNA.

### Influenza A virus induces relocalization of Hsp40 to the nucleus at early stages of infection

To further understand NP-Hsp40 interaction in context of IAV infection, we performed a time course experiment to examine the temporal-subcellular distribution of Hsp40 along with IAV NP in uninfected and IAV infected cells. We observed that in uninfected cells, Hsp40 has diffused localization across nucleus and cytoplasm (Fig. 1d), which is in accordance with earlier reports^41^. However upon IAV infection the subcellular distribution of Hsp40 changed dramatically. At 1 h post-infection (p.i.), Hsp40 co-localized with incoming NP in the plasma membrane proximal region of cytoplasm and subsequently at 4 h p.i., it translocated in to nucleus of infected cells (arrows indicate the co-localization). At later stages (8 h p.i. and 24 h p.i.), NP localization changed to cytoplasmic, however Hsp40 remained nuclear. The distribution of NP during the course of infection was observed to be in conformance with the earlier published reports^42^,^43^. To confirm early stage co-localization of Hsp40 with vRNPs, we employed fluorescence *in situ* hybridization using positive sense RNA probes against NP vRNA to detect IAV vRNP in combination with immunofluorescence (FISH/IF) to detect NP and Hsp40. In line with our time course data, we found Hsp40 was co-localized with NP and NP vRNA in the nucleus at 4 h p.i. (Fig. 1e). Again, at 24 h p.i., the NP vRNA and NP were found to be localized mainly in the cytoplasm while Hsp40 localization remained unchanged. Taken together these data suggest that Hsp40 may associate with incoming vRNPs during early stages of infection and relocalize to nucleus. The dramatic redistribution of Hsp40 observed at early stages of virus infection and its association with parental vRNP suggests it may be recruited by IAV to facilitate the nuclear transport of vRNPs.

### NP-Hsp40 interaction is mediated by the N-terminal domains of both proteins

IAV NP is a 55 KDa protein comprised of 498 amino acids. The primary sequence of NP can be divided into three functional domains: the N-terminal RNA and PB2 binding domain (body), NP-NP homo-oligomerization domain (head) followed by NP-NP homo-oligomerization and PB2 binding domain (body and tail loop) (Fig. S1a)^44^,^45^,^46^,^47^,^48^. The NP interacting partner Hsp40, also known as chaperone DnaJ, consists of 360 amino acids ordered in to two domains: the N-terminal J domain that binds with Hsp70 and C-terminal substrate binding domain^29^ (Fig. S1b). In order to identify the protein domains involved in NP-Hsp40 interaction, we cloned a series of N- and C-terminal 6X His tagged NP deletions and GST tagged Hsp40/DnaJB1 deletions into bacterial expression vectors. We expressed and purified these recombinant proteins in *E.coli*, and carried out *in vitro* GST pull down assays. Immunoblotting of pull down fractions with anti-His antibody to detect NP revealed that apart from full length NP, the N-terminal fragment of NP could also interact with GST-Hsp40/DnaJB1 (Fig. S1c). Next, to define the specific region of Hsp40/DnaJB1 required for interaction with NP, the N-terminal J domain and C domain of Hsp40 were expressed as N-terminal GST tagged proteins in *E.coli*. We observed that along with full length GST-Hsp40, the GST-J domain was also able to pull down full-length NP (Fig. S1d). These studies indicate that interaction between IAV NP and cellular Hsp40 is mediated specifically through the N-terminal J domain of Hsp40 and N-terminal RNA/PB2 binding domain of NP. The NP-Hsp40 interaction has been validated previously in various strains and subtypes including H1N1, H5N1 and H3N2 by Sharma *et al.*, 2011^10^. Interestingly, the sequence alignment of NP protein sequences from different subtypes indicated that the N-terminal region of NP is highly conserved across various subtypes (Fig. S1e), supporting the conserved nature of interaction between Hsp40 and NP.

### Heat shock protein chemical inhibitor KNK437 reduces NP localization to the nucleus in infected cells

**Figure 2:**
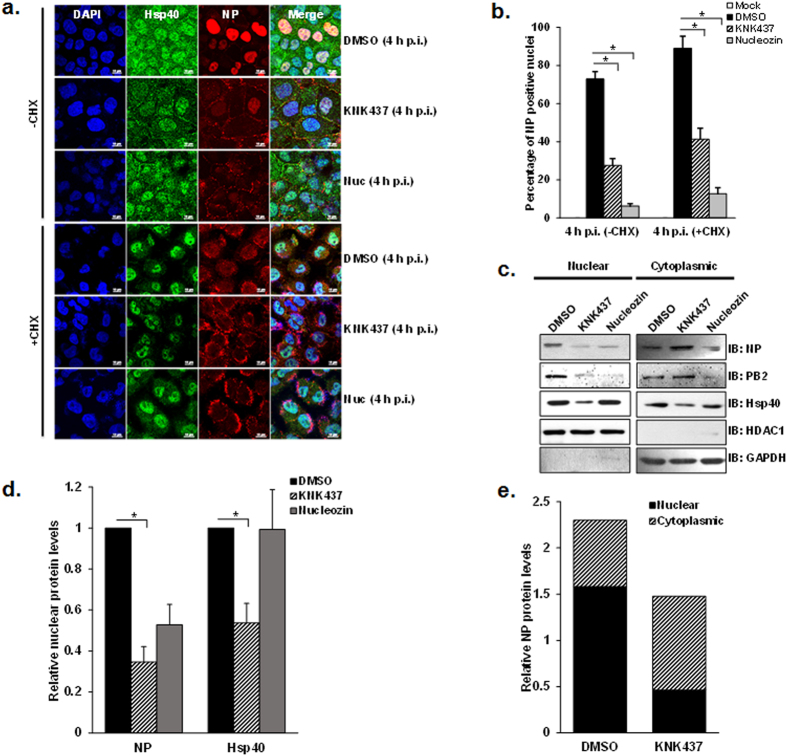
Drug mediated inhibition of Hsp impedes nuclear import of incoming IAV NP in virus infected cells. (**a**) Confocal analysis indicates diminished nuclear accumulation of NP after KNK437 treatment. A549 cells were pretreated with DMSO or KNK437 for 6 h. Cells were infected with PR8 virus at MOI = 5 and treated with DMSO, KNK437 or nucleozin in the presence of cycloheximide (CHX). Cells were fixed at 4 h p.i. and observed under confocal microscope with 60X. Cells were stained using anti-NP and anti-Hsp40 primary antibody, followed by Alexa 594 and Alexa 488 conjugated secondary antibodies respectively. (**b**) Quantitative analysis of nucleoprotein localization in mock infected, DMSO, KNK437 and nucleozin treated −CHX and +CHX cells. The images acquired under confocal microscope at low magnification were used to calculate the percentage of cells with nuclear localized NP among total cell population. The results are mean and standard deviation of four replicates. (**c,d**) Western blot analysis shows decrease in NP levels in nuclear fraction after KNK437 treatment. A549 cells were pretreated with DMSO or KNK437 for 6 h. Cells were infected with X-31 virus at MOI = 5 and treated with DMSO, KNK437 or nucleozin. Cells were harvested 4 h post-infection followed by cellular fractionation and western blotting. Densitometry analysis was performed using ImageJ. NP and Hsp40 levels were normalized with HDAC1 levels and relative nuclear protein levels in KNK437 and nucleozin treated cells with reference to DMSO treated cells were calculated and plotted. (**e**) Nuclear to cytoplasmic ratio of NP protein levels decreases significantly in KNK437 treated cells. Relative NP protein levels in different fractions were calculated by densitometry analysis of western blots after cellular fractionation. Protein levels obtained were normalized by the total protein amount loaded as quantitated by Bradford assay. Data show mean ± S.D. from one representative experiment (n = 3) of at least three independent experiments. Statistical significance was determined using Student’s t test. *p < 0.05.

Association of Hsp40 with incoming vRNPs through NP during early stages of the infection and consecutive translocation of both the proteins to nucleus, suggested a role of Hsp40 in the vRNP nuclear import. To test this possibility, we studied the distribution of NP upon modulating Hsp40 levels in infected cells. For this, we used a chemical inhibitor KNK437, a benzylidene lactam compound known to inhibit the induction of various heat shock proteins including Hsp40, Hsp70, Hsp72 and Hsp105 (see supplementary Fig. S2a)^49^. We investigated the effect of KNK437 on virus replication at different doses at 24 h p.i., and observed reduction of NP protein levels with a corresponding decrease in Hsp40 levels in a drug dose dependent manner, with significant reduction at 200μM concentration (Fig. S2b,c). We checked the effect of KNK437 on cell viability using an MTT assay at this concentration after treating mock infected or virus-infected A549 cells with DMSO or KNK437 for 24 h at 1 MOI (multiplicity of infection). We found no significant cytotoxic effect on the cells at this concentration (p = 0.8, 0.21), therefore later on all experiments were done at 200 μM concentration of KNK437 (Fig. S2d). We used this inhibitor to further study the role of Hsp40 in nuclear translocation of IAV NP by immunofluorescence. To this end, A549 cells were pretreated with DMSO or KNK437 for 6 h followed by infection with PR8 at MOI = 5 for 4 h. In mock treated cells, NP localized exclusively in the nucleus at 4 h p.i., however, in KNK437 treated cells, punctate distribution of NP was observed in the cytoplasm with significantly reduced localization in the nucleus. We used nucleozin treatment as positive control in this assay, which results in aggregation of NP, hence inhibiting its nuclear translocation^50^. To distinguish between incoming vRNPs and neo-synthesized vRNPs, we performed a similar immunolabeling experiment with the cells infected in the presence of cycloheximide. Similar retention of NP as cytoplasmic punctate was observed in cycloheximide treated cells. Representative results of the localization of NP and Hsp40 are shown in Fig. 2a. The immunostained cells were counted and intracellular localization pattern of NP was analyzed using NIS-Elements AR software as described in the methods. Nuclear localization of NP was found to be significantly reduced upon KNK437 pretreatment, in both CHX +ve (~54% reduction) and CHX −ve cells (~62% reduction) to similar levels. In comparison NP nuclear localization was drastically reduced in nucleozin treated CHX +ve (~86% reduction) and CHX −ve cells (~91% reduction) (Fig. 2b) (p = 0.0005 and 0.01; 0.00003 and 0.004). To further validate the effect of Hsp down-regulation on NP localization, we determined the distribution of NP and Hsp40 by cellular fractionation and western blot analysis. We also checked the distribution of the PB2 subunit of the polymerase complex in different subcellular fractions for comparison. By this method also nuclear localization of NP was found to be significantly reduced along with PB2, upon KNK437 treatment (Fig. 2c,d). The purity of fractions was confirmed by immunoblotting for HDAC1 (nuclear marker) and GAPDH (cytoplasmic marker). Further, the relative NP protein levels were quantified in nuclear and cytoplasmic fractions by densitometry, and nuclear/cytoplasmic ratio of NP was found to be significantly reduced upon KNK437 treatment (Fig. 2e). Collectively, these results indicated that KNK437 interferes with nuclear import of NP during early stages of infection.

### KNK437 inhibits the propagation of influenza A viruses

**Figure 3:**
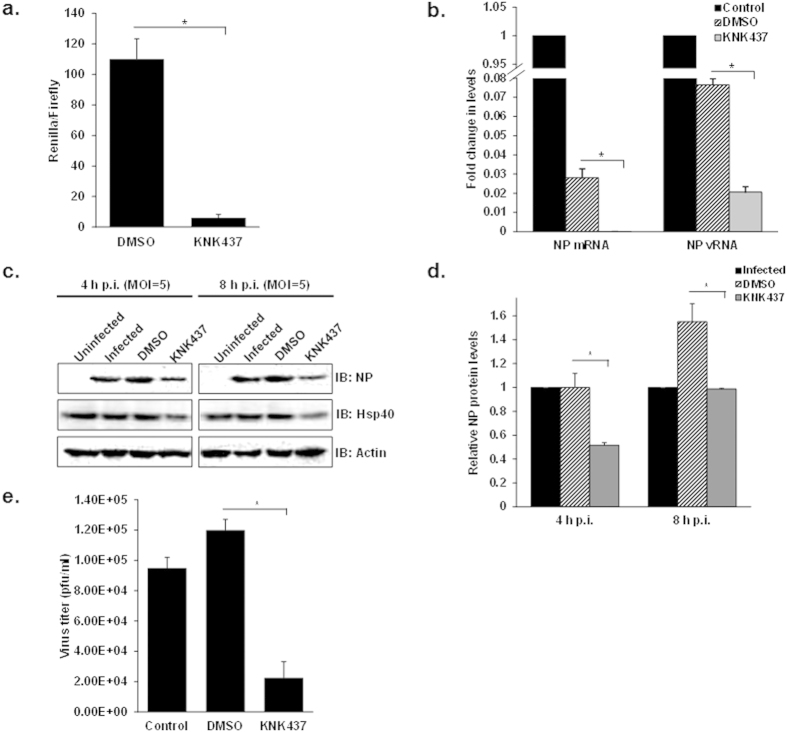
Hsp inhibitor KNK437 impairs IAV polymerase function, viral protein accumulation and overall replication. (**a**) KNK437 significantly reduces RNP activity. A549 cells were transfected with plasmids containing PA, PB1, PB2, NP, NS1-Renilla luc/pPolI derived from A/Puerto Rico/8/1934 H1N1 along with internal transfection control plasmid pGL3 constitutively expressing Firefly luciferase. Cells were treated with 200 μM KNK437 at the time of transfection and relative IAV Pol activity was measured by taking ratio renilla/firefly luc counts. (**b**) KNK437 mediated Hsp inhibition reduces NP mRNA and vRNA levels. A549 cells were pretreated with DMSO or 200 μM KNK437 for 6 h followed by infection with IAV WSN at MOI = 5. NP mRNA and vRNA levels were quantified by qRT-PCR at 4 h p.i. in DMSO or KNK437 treated cells. The fold change in levels was calculated with reference to control infected cells. (**c**) Hsp inhibition significantly impairs viral protein accumulation. A549 cells were pretreated with DMSO or 200 μM KNK437 for 6 h followed by infection with PR8 virus at MOI = 5. Cells were harvested at 4 h and 8 h p.i. and cell lysate was subjected to SDS-PAGE followed by western blotting to check viral protein levels. (**d**) Densitometry analysis was done using ImageJ and NP levels normalized against actin were plotted on the graph. (**e**) KNK significantly impairs virus replication. Supernatant (cell media) was collected at 24 h p.i. to perform plaque assay. Data show mean ± S.D. from one representative experiment (n = 3) of at least three independent experiments. Statistical significance was determined using Student’s t test. *p < 0.05.

IAV replicates its genome in the nucleus of the host cell, and any defect in the nuclear import of its genome should reflect in reduced polymerase activity. To confirm Hsp40’s role in this process, we investigated the effect of KNK437 on the activity of viral polymerase complex using a luciferase reporter based influenza mini-genome assay^51^. For this, A549 cells were treated with DMSO or KNK437, followed by co-transfection with plasmids encoding A/Puerto Rico/8/1934 H1N1 derived PA, PB1, PB2, NP and reporter plasmid containing untranslated region of NS1 segment upstream of luciferase gene. Polymerase-driven luciferase activity was measured 24 h post transfection. As predicted, KNK437 strongly inhibited the viral polymerase- activity (~10 folds), without affecting the expression of the control reporter plasmid (Fig. 3a) (p = 0.001). The decrease in viral polymerase activity in KNK437 treated cells could be due to a decrease in the nuclear accumulation of viral polymerase proteins or non-functional polymerase complex formation in the nucleus. To further confirm impaired IAV polymerase function in KNK437 treated cells, we measured NP vRNA and mRNA levels in IAV infected cells. To this end, A549 cells were pre-treated with DMSO and KNK437, followed by infection with PR8 virus at MOI = 5. Total RNA was isolated and subjected to quantitative RT-PCR to measure viral NP vRNA and mRNA. To avoid nonspecific quantification among different types of viral RNA, we employed the methods described by Kawakami *et al.*^52^. This improved method of quantification involves a hot-start strand-specific reverse transcription with tagged primers and trehalose, allowing strand specific quantification of viral mRNA and vRNA. As expected NP mRNA and vRNA levels were found to be significantly reduced in KNK437 treated cells as compared to DMSO control (p = 0.0005, 0.005) (Fig. 3b). Next we assessed the effect of Hsp inhibitor on accumulation of viral protein in IAV infected cells. Viral NP levels were found to be significantly lower in KNK437 treated cells as compared to the DMSO treated or untreated cells after single cycle of infection (Fig. 3c,d). Further, measurement of virus titers by plaque assay was conducted to test the effect on overall IAV replication. KNK437 treated cells displayed 80% reduction in virus titers as compared to DMSO control at 24 h p.i. (p = 0.02) (Fig. 3e). We also noticed that viral titers were higher in cells treated with DMSO as compared to untreated cells. These results are consistent with previously published report that the yield of infectious virus is enhanced at intermediate concentrations of DMSO^53^. Taken together, these results indicated that the Hsp inhibition by KNK437 markedly reduces viral transcription, protein accumulation, and overall replication and this reduction is not related to the cytotoxicity of the inhibitor. We also studied the inhibitory effect of KNK437 in cells infected with different viral isolates (A/Puerto Rico/8/1934 H1N1, A/X-31 H3N2, A/WSN/1933 H1N1, A/California/08/2009 H1N1) (see supplementary Fig. S3) and in different cellular backgrounds (human lung adenocarcinoma A549, canine kidney-derived MDCK and normal human bronchial epithelial NHBE primary cells) (see supplementary Fig. S4) by assessing the NP protein levels and virus titer at 24 h p.i. The effect of KNK437 was found to be conserved among different influenza A viruses and IAV-permissive cell lines indicating broad spectrum inhibitory effect of KNK437 on influenza virus replication.

### RNAi-mediated knockdown of endogenous Hsp40 reduces nuclear accumulation of incoming IAV NP

**Figure 4:**
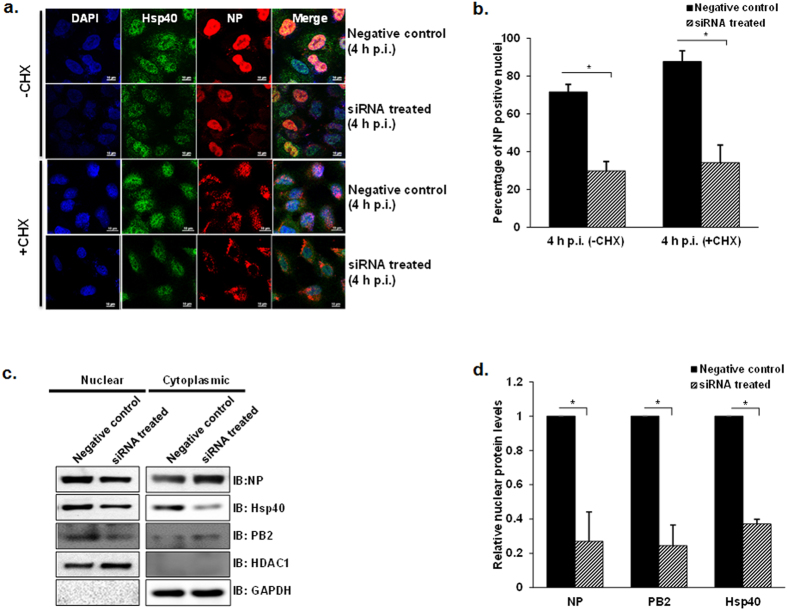
Hsp40 specific RNAi inhibits nuclear import of IAV NP in virus infected cells. (**a**) Confocal microscopy indicates decreased NP translocation to nuclear on Hsp40 knockdown. A549 cells were transfected with pooled siRNA against Hsp40/DnaJB1 and control non-targeting siRNA. 24 h post-transfection, cells were infected with X-31 virus at MOI = 5 in the presence of cycloheximide. Cells were fixed 4 h post-infection followed by immunostaining for NP (red) and Hsp40 (green). **(b)** Quantitative analysis of nucleoprotein localization in non-targeting siRNA treated and Hsp40 siRNA treated −CHX and +CHX cells. The images were acquired under confocal microscope with 40X objective lens and percentage of cells with nuclear localized NP among total cells was calculated. The results are mean and standard deviation of four replicates. **(c,d)** Western blot analysis further confirms the effect of Hsp40 knockdown on NP and PB2 localization. From the similar experiment as shown in Fig. 4a, cells were harvested 4 h post-infection and subjected to cellular fractionation followed by western blotting. HDAC1 and GAPDH were used as loading control for nuclear fraction and cytoplasmic fraction respectively. Densitometry analysis was performed using ImageJ. NP, PB2 and Hsp40 levels in nuclear fraction were normalized with HDAC1 levels and relative nuclear protein levels in siRNA treated cells with reference to control siRNA treated cells were calculated and plotted. Data show mean ± S.D. from one representative experiment (n = 3) of at least three independent experiments. Statistical significance was determined using Student’s t test. *p < 0.05.

**Figure 5:**
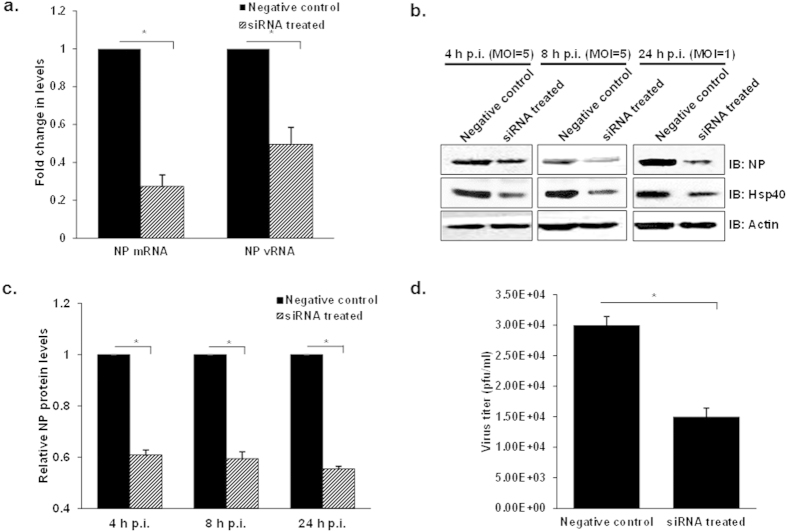
RNAi mediated knockdown of Hsp40 attenuates IAV transcription, viral protein accumulation and replication. (**a**) Down-regulation of Hsp40 significantly reduces NP mRNA and vRNA levels. A549 cells were transfected with non-targeting siRNA or Hsp40-specific siRNA for 24 h followed by infection with IAV WSN at MOI = 5. NP mRNA and vRNA levels were quantified by qRT-PCR in non-targeting siRNA treated and Hsp40 siRNA treated cells at 4 h post-infection. (**b–d**) Hsp40 specific RNAi significantly modulates virus translation and replication. A549 cells were transfected with siRNA against Hsp40/DnaJB1 or non-targeting siRNA. 24 h post-transfection cells were infected with PR8 virus at MOI = 5 (4, 8 h) and 1 (24 h). Cell lysates harvested at different time points post-infection were subjected to western blotting to check NP and Hsp40 levels (**b,c**). The fold change in NP mRNA or vRNA levels and relative protein levels were calculated with reference to non-targeting siRNA treated cells. Culture media collected at 24 h p.i. was used to carry out plaque assay (**d**). Data show mean ± S.D. from one representative experiment (n = 3) of at least three independent experiments. Statistical significance was determined using Student’s t test. *p < 0.05.

Since KNK437 is not an exclusive inhibitor of Hsp40, therefore to rule out its non-specific effect on IAV replication we used Hsp40/DnaJB1-specific small interfering RNA (siRNAs) to knock down constitutively expressed Hsp40. The efficacy of siRNA against Hsp40/DnaJB1 was characterized at different doses (100 nM, 150 nM and 200 nM) in infected A549 cells. The levels of endogenous Hsp40 significantly decreased after transfecting SMART pool siRNA against Hsp40 as compared to the untreated cells (see supplementary Fig. S5). However, the levels of Hsp40 did not change considerably at higher siRNA concentration; therefore 100nM concentration was used for further experiments. Further, we assessed the effect of siRNA-mediated Hsp40 knockdown on nuclear localization of NP. For this, A549 cells were transfected with 100 nM siRNA against Hsp40 or control siRNA for 24 h followed by infection with IAV in the presence of cycloheximide. As compared with the control siRNA transfected cells, Hsp40 siRNA transfected cells showed decreased nuclear translocation of NP as observed by immunofluorescence analysis in both CHX treated and untreated cells (Fig. 4a). Quantitative analysis of confocal images was done to calculate the percentage of cells with nuclear NP using NIS-Elements AR software. Knockdown of Hsp40 decreased the proportion of cells with predominantly nuclear localization of NP by more than 50% as compared to control siRNA-treated cells (p = 0.0006; 0.02) (Fig. 4b).To further confirm this observation, NP, PB2 and Hsp40 levels were checked by western blot analysis in nuclear and cytoplasmic fractions. NP and PB2 levels were found to be reduced in the nuclear fraction in Hsp40 siRNA treated cells, confirming that Hsp40 knockdown reduces IAV NP and RNP accumulation in the nucleus (Fig. 4c,d). These results show that endogenous Hsp40 plays a key role in the nuclear localization of parental IAV RNP. Since siRNA mediated knockdown of Hsp40 significantly reduced nuclear accumulation of IAV NP, we expected that virus transcription, protein accumulation and replication would similarly be affected. To test this, we monitored NP vRNA and mRNA levels in A549 cells transfected with Hsp40 siRNA or control siRNA for 24 h followed by IAV infection. The NP mRNA and vRNA levels were found to be significantly reduced in siRNA treated cells at 4 h p.i. (p = 0.003 and 0.01) (Fig. 5a). We further monitored NP protein levels in siRNA treated cells after single and multiple cycles of infection. Immunoblot analysis showed that the NP levels decreased dramatically in Hsp40/DnaJB1 siRNA treated cells at different time points post-infection (Fig. 5b,c). As expected, IAV replication was also reduced in Hsp40-siRNA treated cells as compared to the control siRNA (p = 0.01) (Fig. 5d) Together, these observations confirmed that down-regulation of Hsp40 expression specifically results in the reduction of IAV NP nuclear import and hence viral replication.

### Hsp40 over-expression enhances nuclear localization of NP

**Figure 6:**
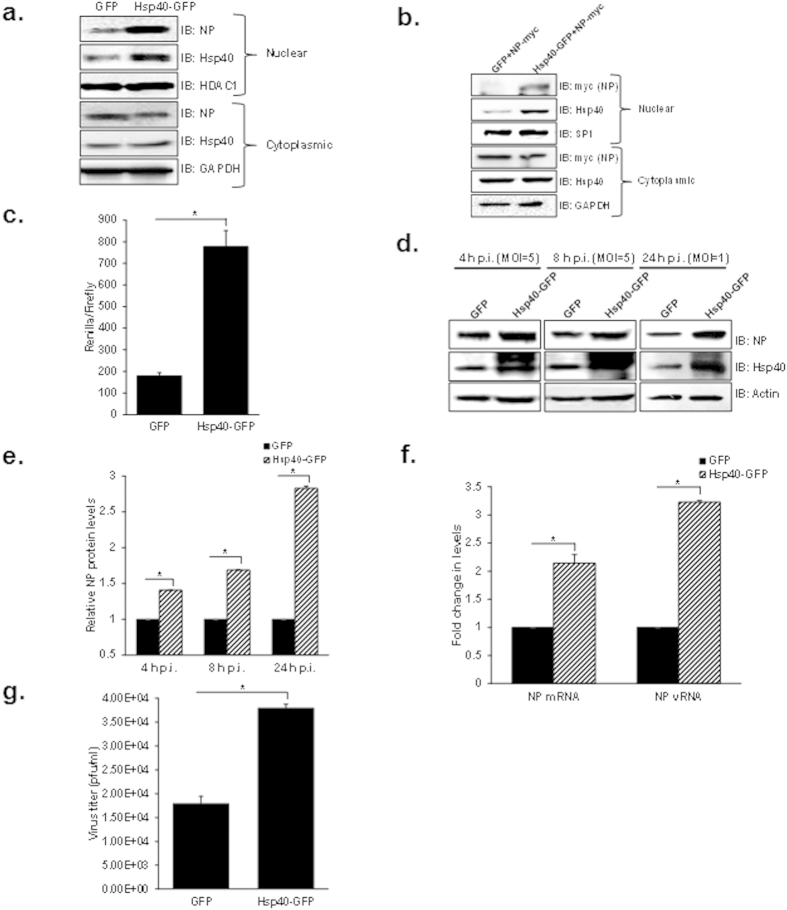
Over-expression of Hsp40/DnaJB1 promotes nuclear accumulation of IAV NP and enhances IAV replication. (**a**) IAV NP nuclear distribution increases on up-regulating Hsp40/DnaJB1 levels. A549 cells were transfected with GFP or Hsp40-GFP. 24 h post-transfection, cells were infected with PR8 virus at MOI = 5. Cells were harvested 4 h post-infection, subjected to cellular fractionation followed by western blotting. HDAC1 and GAPDH were used as loading control for nuclear and cytoplasmic fractions respectively. (**b)** Over-expression of Hsp40 enhances the nuclear localization of transiently expressed NP. HEK293 cells were co-transfected with GFP or Hsp40-GFP and NP-pCDNA. 48 h post-transfection, cells were harvested and subjected to cellular fractionation followed by western blotting. SP1 and GAPDH were used as loading control for nuclear and cytoplasmic fractions respectively. **(c)** Over-expression of Hsp40 significantly enhances RNP activity. A549 cells were transfected with plasmids containing PA, PB1, PB2, NP and NS1-luc/pPolI derived from A/Puerto Rico/8/1934 H1N1 and internal transfection control plasmid pGL3 along with GFP or Hsp40-GFP. Cells were harvested 24 h post-transfection and effect on polymerase activity was determined by measuring change in luciferase activity. (**d–g**) A549 cells were transfected with control plasmid or Hsp40-GFP. 24 h post-transfection, cells were infected with virus at MOI = 5 (4 and 8 h) and 1 (24 h). Cells were harvested at different time points post-infection to perform western blotting (**d,e**) and qRT-PCR to check NP mRNA and vRNA levels at 4 h p.i. (**f**). Cell media was collected at 24 h p.i. to carry out plaque assay (**g**). The fold change in NP mRNA or vRNA levels and relative protein levels were calculated with reference to GFP transfected cells. Data show mean ± S.D. from one representative experiment (n = 3) of at least three independent experiments. Statistical significance was determined using Student’s t test. *p < 0.05.

To further validate the role of Hsp40 in IAV replication, we sought to determine whether the over-expression of Hsp40/DnaJB1 would alter NP cellular localization and affect IAV replication. For this, A549 cells were transfected with Hsp40/DnaJB1 expression plasmid and 24 h post-transfection infected with IAV. Cells were harvested 4 h post-infection and subjected to cellular fractionation followed by western blot analysis. NP levels were found to be enhanced in nuclear fraction in Hsp40 transfected cells as compared to vector control (Fig. 6a). We also checked the effect of Hsp40 over-expression on localization of transiently expressed NP. For this, HEK cells were co-transfected with NP-pCDNA and Hsp40-GFP or GFP expression plasmids. 48 h post-transfection, cells were harvested and NP and Hsp40 levels were checked in nuclear and cytoplasmic fractions by western blotting. Interestingly, co-transfection of NP-pCDNA and Hsp40-GFP increased the nuclear localization of NP (Fig. 6b). Further, we checked the effect of Hsp40 overexpression on IAV polymerase activity by minigenome assay. It was found to be significantly increased upon Hsp40 over-expression (p = 0.007) (Fig. 6c).

We also studied the effect of over-expression of Hsp40 on influenza virus replication. A549 cells were transfected with Hsp40-GFP or GFP for 24 h and then infected with X31 IAV. Cells were harvested at different time points and cell lysate was subjected to western blotting to check the levels of NP and Hsp40. We found NP levels to be up-regulated upon over-expression of Hsp40/DnaJB1 (Fig. 6d,e). We also checked the levels of NP mRNA and vRNA at 4 h p.i. by qRT-PCR and the levels were found to be significantly increased on over-expression of Hsp40 (p = 0.008, 0.0001) (Fig. 6f). Further, cell supernatants collected at 24 h p.i. from the same experiment was used to measure the virus titers. It was observed that up-regulation of Hsp40 enhances virus replication (p = 0.013) (Fig. 6g). Taken together, the data above clearly demonstrate that Hsp40/DnaJB1 is a pro-viral factor for IAV and it promotes virus replication by assisting nuclear import of IAV RNP.

### Hsp40 knockdown impairs efficient IAV NP-importin alpha interaction during IAV infection

**Figure 7:**
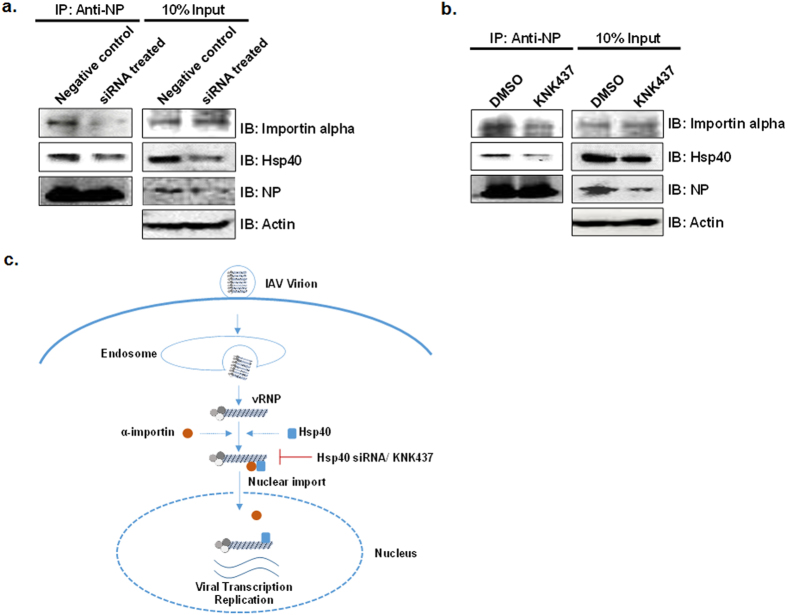
Hsp40 knockdown impairs IAV NP-importin alpha interaction during IAV infection. **(a,b**) Hsp40 inhibition significantly reduced NP-importin alpha interaction. A549 cells were transfected with Hsp40 specific siRNA or control siRNA for 24 h (**a**) or cells were pre-treated with DMSO or KNK437 for 6 h (**b**). Cells were infected with IAV at MOI = 10 for 4 h followed by IP using anti-NP antibody. Hsp40 and importin-alpha levels were checked in immunoprecipitate by western blotting. **(c)** Proposed model of NP nuclear import. NP component of incoming vRNPs associates with Hsp40, along with importin alpha and the entire complex is then translocated to the nucleus. Hsp40 inhibition using chemical inhibitor KNK437 or specific siRNA inhibits NP-importin alpha association and hence reduces IAV NP nuclear translocation.

IAV NP exploits classical pathway of nuclear import by binding to alpha importins through its nuclear localization signals^23^. It was interesting to study whether Hsp40/DnaJB1 is involved in NP-importin alpha interaction and subsequent nuclear import. To test this, we transfected A549 cells with siRNA pool targeting Hsp40 for 24 h, infected them with IAV for 4 h and performed immunoprecipitation using anti-NP antibody to check the status of interaction between NP and importin alpha. Interestingly, the co-immunoprecipitated levels of importin alpha were reduced in Hsp40 siRNA treated cells (Fig. 7a). Similar reduction in NP-alpha importin binding was observed upon treatment of cells with KNK437 before infection (Fig. 7b). These results indicate that Hsp40/DnaJB1 may facilitate the interaction between NP and importin alpha, which is essential for nuclear import of IAV vRNPs. Considering the data on nuclear import of IAV NP and possible role of Hsp40 in and supporting the NP-importin interaction, we proposed a model for proviral action of Hsp40 in IAV replication, shown in Fig 7c.

## Discussion

Establishment of productive influenza A virus infection involves interaction of multiple host cellular factors with viral proteins and is also accompanied by dynamic changes in the cellular gene expression and protein localization. IAV RNA is transcribed and replicated in the nucleus and nucleocytoplasmic shuttling of vRNPs is a crucial process in virus replication. At early stages of infection incoming vRNPs quickly translocate to the nucleus to initiate transcription of viral genes. Consecutively new NP is translated from viral mRNAs in the cytoplasm and transported to nucleus to spur the process of transcription and replication of viral genome^54^,^55^. At late stages of infection the vRNPs are exported out of nucleus and NP starts to accumulate in the cytoplasm^28^,^56^,^57^. This temporal change in the localization of NP is a tightly regulated process and requires involvement of several viral and cellular factors^22^,^23^,^28^,^58^,^59^. Cellular importins have been implicated in nuclear import of IAV vRNPs through interaction with NLS of IAV NP and polymerase components; however it’s likely that this process may be assisted by more host factors^23^,^25^. In this study we have done functional characterization of a previously reported interaction of IAV NP with cellular Hsp40 and discovered that Hsp40/DnaJB1 assists IAV vRNP nuclear trafficking through its interaction with nucleoprotein. Our preliminary studies to identify the biological significance of the NP-Hsp40 interaction showed that Hsp40 associates with incoming vRNPs through nucleoprotein, early on during infection. We also observed that during IAV infection, incoming NP co-localized with the Hsp40 as punctate foci in the cell membrane proximal region of the cytoplasm (1 h p.i.) and very soon both the proteins acquired primarily nuclear localization (4 h p.i.). The simultaneous change in NP and Hsp40 localization to nucleus along with the evidence for interaction between Hsp40 and IAV RNP suggested that NP and Hsp40 may form a complex in the cytoplasm prior to being translocated together into the nucleus. Interestingly, Hsp40 has been reported to facilitate nuclear translocation of other viral and cellular proteins^30^,^60^,^61^. To test whether Hsp40 plays a similar role during IAV infection, we used siRNAs and small molecule chemical inhibitor KNK437 to down-regulate Hsp40 and studied its effect on vRNP nuclear import and IAV replication. We observed that both RNAi and chemical inhibition of Hsp40 reduced cytoplasmic to nuclear translocation of incoming NP, which subsequently resulted in decreased viral polymerase activity, reduced transcription, protein accumulation and attenuated overall IAV replication. The pro-viral function of Hsp40 was further confirmed by Hsp40/DnaJB1 overexpression which enhanced viral gene expression and virus replication.

We mapped the interacting domains of NP and Hsp40 and observed that N-terminal regions of both NP and Hsp40 are required and sufficient for NP-Hsp40 interaction. Further, using bioinformatics tool cNLS mapper^62^ we predicted Hsp40 NLSs to be specific for importin αβ pathway, the same used by IAV NP for nuclear translocation. It has been predicted that Hsp40 contains NLS sequence in its C-terminal region (176 a.a. to 186 a.a.) (Prediction score = 6.5). Interestingly we observed that down-regulation of Hsp40 through KNK437 or specific siRNAs coincides with reduced interaction between NP and importin alpha. On the basis of these observations we proposed a likely mechanism where Hsp40 may bind to IAV NP through its J domain and recruit cellular importins through its C-terminus thereby facilitating NP-importin interaction and importin mediated nuclear import of vRNPs. However this model requires further characterization and formation of a ternary complex between IAV NP, Hsp40 and importin alpha needs to be biochemically validated. Recently, Cao *et al.*, reported that another Hsp40 family member DnaJA1 associates with influenza viral polymerase subunits PB2 and PA through its C-terminal substrate binding domain and enhances its polymerase activity^63^. Although this study corroborates our observation that Hsp40 is a positive regulator of IAV polymerase activity, it does not show interaction between Hsp40 and incoming vRNPs. Moreover, they attribute the enhanced polymerase activity to PB2 and PA, instead of NP translocation to nucleus.

The small molecule Hsp inhibitor KNK437 used in our study is a benzylidene lactam compound with limited toxicity and has been suggested as an effective chemo-sensitizer for cancer therapy^64^,^65^. The inhibitory effect of KNK437 on IAV replication was found to be consistent in various cell types and IAV strains suggesting Hsp40 requirement to be a conserved feature. In addition to IAV, Hsp40 has also been reported to be required for efficient replication for HIV, HBV and CMV^9^,^30^,^60^. This projects KNK437 as a potential broad spectrum antiviral against IAV and other important viral pathogens. Overall, the present study extends the knowledge on cellular chaperones involved in influenza virus replication and identifies Hsp40 as a promising candidate to develop anti-influenza modalities.

## Methods

### Cell lines, Plasmids and Antibodies

Human lung adenocarcinoma (A549) cells, Madin-Darby canine kidney (MDCK) epithelial cells, and human embryonic kidney 293 (HEK293) cells obtained from ATCC (Manassas, VA, USA) were cultured in DMEM medium (Hyclone, UT, USA) supplemented with 10% fetal calf serum (Hyclone), 100 units/ml Penicillin Streptomycin (Invitrogen, NY, USA). Normal human bronchial epithelial (NHBE) cells were purchased from Lonza, Switzerland and grown in BEGM medium. The NP gene of A/Chicken/Hatay/2004 (H5N1) influenza virus was cloned in pCDNA3.1 myc- vector (Invitrogen), pET-28(+) vector (Novagen, MA, USA). DNAJB1 (Hsp40) (NM_006145) cloned in pCMV6-AC-GFP vector was purchased from Origene (MD, USA). For Hsp40 domain mapping, a full length gene, J domain and C domain were cloned in pGEX4T1 vector (GE healthcare, Sweden) to express recombinant protein with N-terminus GST tag. The antibodies used in the study include anti-NP mouse monoclonal (obtained from Immunology and Pathogenesis Branch, Influenza Division, CDC, Atlanta, GA, USA), anti-Hsp40 rabbit polyclonal (Cell Signaling, MA, USA), anti-HDAC1 rabbit polyclonal (Santa Cruz, Texas, USA), anti-GAPDH mouse monoclonal (Sigma Aldrich, MO), anti-actin mouse monoclonal (Sigma Aldrich), anti-karyopherin-α1 (anti-importin alpha) mouse (Santa Cruz).

### Transfection, IAV infection and plaque assay

DNA transfections were done using Lipofectamine 2000 (Invitrogen) in HEK293 cells maintained in DMEM medium without serum and antibiotic. Culture medium was replaced with DMEM medium containing 10% FCS after 4 h. For infection, 90–100% confluent cells were washed with PBS. All virus infections were done in DMEM medium containing 0.3% BSA (GIBCO, NY, USA). Cells were infected with virus at different multiplicity of infection as mentioned. After virus adsorption for 1 h, cells were washed with PBS and incubated with DMEM medium containing 0.3% BSA and 1 μg/ml N-p-tosyl-1-phenyl alanine chloromethyl ketone (TPCK) (Sigma Aldrich). For plaque assays, MDCK cell monolayers seeded in 6-well plates were washed with PBS and 200 μl culture supernatants of infected cells was added at different dilutions. After virus adsorption for 1 h, cells were washed with PBS and overlaid with 1.6% Agarose (SeaKem LE, USA) in L15 medium (2×L15, 1 M HEPES, 200 mM Glutamine, 50 μg/ml Gentamycin, NaHCO_3_ and Penicillin Streptomycin) containing Trypsin-TPCK (Sigma Aldrich). The plates were incubated for 48 to 72 h at 37 °C, agarose was removed, and cells were fixed with 70% ethanol and stained with crystal violet. Cells were washed with distilled water, dried and plaques were counted.

### KNK437 and cycloheximide treatment

KNK437 and cycloheximide were purchased from Calbiochem (Darmstadt, Germany). For all experiments, A549 cells were pre-treated with DMSO or 200 μM KNK437 for 6 h. Cells were then infected with virus followed by treatment with DMSO or KNK437 in infection medium. Cells were treated with 100 μg/ml cycloheximide 1 h post-infection.

### Nuclear cytoplasmic extraction and immunoblotting

Cells were harvested in ice-cold PBS and collected by centrifugation at 211 *g* for 5 min at 4 °C. Nuclear cytoplasmic fractionation was done using the Active Motif nuclear extract kit (CA, USA). Briefly, cells were resuspended in hypotonic buffer and incubated for 15 min at 4 °C followed by addition of detergent. Cytoplasmic fraction was collected by centrifugation at 14,000 *g* for 30 sec. Nuclear pellet was washed with PBS and resuspended in complete lysis buffer. Nuclear debris was removed by centrifugation at 15,000 *g* for 15 min and supernatant was collected as nuclear fraction. The proteins in different fractions were separated on SDS-PAGE followed by western blotting. The proteins in the nuclear and cytoplasmic fractions were analyzed using anti-HDAC1 or anti-SP1 and anti-GAPDH antibodies respectively as loading control.

### Immunoprecipitation and RNase treatmen**t**

A549 cells infected with PR8 virus were harvested at appropriate time point post-infection and resuspended in 400 μl non-denaturing lysis buffer (20 mM Tris-Cl pH 8.0, 137 mM NaCl, 10% glycerol, 0.5% NP-40, 2 mM EDTA). After sonication, homogenates were centrifuged at 15870 *g* for 15 min. The supernatants were used to set up immunoprecipitation assay. For RNase treatment, 100μg/ml RNase was added to cell lysate and mix was incubated on ice for 45 min. Cell extracts were mixed with appropriate antibody and incubated at 4 °C for overnight. Next day, protein G dynabeads (Invitrogen) were mixed and rotated at 4 °C for 2 h. The beads were washed thrice with PBS and protein-antibody complexes were eluted in 2X Laemmelli buffer. The immunoprecipitated proteins were separated on SDS-PAGE followed by western blotting. The remaining cell lysate was treated with proteinase K (Thermo Fisher Scientific) followed by RNA isolation using acidic phenol:chloroform:isoamyl alcohol mix. Purified RNA was analyzed on formaldehyde agarose gel.

### Immunofluorescence microscopy

A549 or HEK293 cells were grown on coverslips. After transfection or infection, cells were fixed with 4% paraformaldehyde. Cells were washed with PBS and blocked with 2% BSA in PBS. Coverslips were incubated with specific antibodies for Hsp40 and NP at 4 °C overnight. After washing with PBS, cells were subsequently incubated with indicated Alexa Fluor (Invitrogen) conjugated secondary antibodies for 2 h at RT. After washing, cells were mounted in antifade (with DAPI) on slides and images were acquired using confocal microscope (Nikon, Japan) and analyzed using NIS-Elements.

### Quantitation of nuclear NP-positive cells

For scoring, all the images were acquired using confocal microscope at 40X magnification with same parameters. The total number of cells were determined by scoring the number of nuclei (stained with DAPI) using NIS-Elements AR software. The average fluorescence intensity of Alexa 594 in nuclei of each cell was determined using binary to ROI and ROI statistics function. The cell with average fluorescence intensity value more than the threshold value was scored as positive nuclear NP and then percentage of cells having positive nuclear NP signal among the total cell population was calculated. The scoring was done for at least 5 different fields and the mean value was plotted.

### Fluorescence *in situ* hybridization

Positive sense RNA probes were produced using linearized pCDNA-NP as template, for *in vitro* transcription using digoxigenin (DIG) RNA labelling kit (Roche, Indianapolis). Probes were ethanol precipitated and resuspended in DEPC water. For immunofluorescence and FISH analysis, A549 cells seeded on coverslips were infected with PR8 virus at MOI = 5. At different time points post infection, cells were fixed with 4% paraformaldehyde and permeabalized with 0.5% triton X-100. For immunofluorescence, cells were blocked with 1% BSA followed by primary and secondary antibodies treatment. The cells were then subjected to another fixation step with 4% paraformaldehyde for 10 min. Cells were washed twice with 1XPBS and equilibrated with 10% formamide 2X SSC for 10 min before ISH procedure. To detect viral RNAs, 0.5 μg/ml of labeled probes were mixed in hybridization buffer. Hybridization was carried out in humidified chambers at 37 °C for 16 h. The cells were washed with 2X SSC buffer at RT and twice at 40 °C. Cells were then incubated in blocking solution containing normal sheep serum in TBST, pH 8.0 for 30 min. The cells were subjected to anti-digoxigenin (DIG) antibody (Roche) treatment followed by staining with TSA Plus Cy3 system (Perkin Elmer). Images were acquired using confocal microscope (Nikon, Japan).

### RNA isolation and quantitative Reverse transcription-PCR

A549 cells were infected with IAV WSN virus at MOI = 5 and cells were harvested at 4 h p.i. RNA was extracted from cells using Trizol reagent (Invitrogen). The cDNA was made using the hot start based Revert Aid Reverse transcriptase enzyme (Thermo Scientific, MA, USA) method described by Kawakami *et al.*, 2011^52^. For NP mRNA and NP vRNA quantification, specific vRNA and mRNA tagged primers were used for cDNA synthesis. A 5.5 μl mixture containing around 500 ng of total RNA and 10 pmol of tagged primer were heated for 65 °C for 10 mins, chilled on ice for 5 mins followed by heating again at 60 °C for 5 mins. To this, 14.5 μl of reaction mixture [5X first strand buffer, 1 μl 0.1 M DTT, 1 μl 10 mM dNTP mix and 1 μl Revert Aid Reverse transcriptase, 1 μl RNasin Plus RNase inhibitor (40 U/μl) (Millipore) and 6.5 μl saturated trehalose] was added and incubated at 60 °C for 1 h. For quantification of actin levels, cDNA was prepared using oligo dT primer. Real time PCR was performed using Syber Green (Thermo Scientific) and expression levels of NP vRNA and mRNA and actin were analyzed. The primers used in this study are listed in Table 1. For quantification of NP vRNA levels, vRNA tag and NP WSN reverse primers were used and for NP mRNA, mRNA tag and NP WSN forward primers were used. The amplification was performed using one cycle of 95 °C for 10 min and 40 cycles of 95 °C for 30 sec, 60 °C for 30 sec and 72 °C for 30 sec followed by melt curve analysis. The changes in threshold cycle (C_T_) values and fold difference was calculated and plotted.

### RNA immunoprecipitation

A549 cells infected with PR8 virus were harvested 4 h p.i. RNA IP assay was performed using Magna RIP kit (Millipore). Briefly, cell pellet was resuspended in RIP lysis buffer. The cleared lysate was used to set up IP reaction using anti-NP, anti-Hsp40 antibodies and rabbit and mouse IgG. The reaction mix was incubated for 1 h at RT followed by addition of magnetic A/G beads and was kept on rotator for overnight at 4 °C. Magnetic beads-bound complexes were immobilized with magnet and unbound materials were washed off. Bound protein was digested using SDS and proteinase K followed by purification of RNA by phenol:choloroform. Purified RNA was used to analyze NP vRNA levels by qRT-PCR using specific primers^55^. The fold change in NP vRNA levels with reference to that of control antibodies was calculated from C_T_ values and the data was plotted with logarithmic scale.

### Gene silencing using siRNA

A549 cells were transfected with 100 nM control non-targeting siRNA pool (Mission siRNA Universal negative control 1, Sigma) or SMART pool siRNA against Hsp40/DnaJB1 (Dharmacon, CO, USA) using Transfection reagent 1 (Dharmacon). The siRNA transfection mix was made in Opti-MEM medium (Life Technologies). Serum containing DMEM medium was added to the cells 6 h p.t. Cells were infected 24 h post-siRNA transfection.

### GST pull down

*E.coli* Rosetta (DE3) cells expressing His-tagged full length NP or various NP deletions were induced with IPTG. Cells were harvested in bacterial lysis buffer (300 mM NaCl, 50 mM NaH_2_PO_4_ pH 7.5 supplemented with 1 mg/ml lysozyme and 1 mM PMSF). Cell suspension was sonicated and lysates were centrifuged at 15870 *g* for 1 h. Supernatant (soluble fraction) was used for purification of protein using Ni-NTA beads (Qiagen, Limburg, Netherlands). For GST pull down, soluble fraction containing either GST or Hsp40-GST were incubated with glutathione sepharose beads (GST sepharose 4 Fast Flow, GE healthcare). After washing, the beads were incubated with purified His-tagged proteins. Beads were washed with lysis buffer and protein complexes were eluted in 1 M Tris-Cl buffer pH 8.0 containing 10 mM reduced glutathione. The protein complexes were resolved on SDS-PAGE followed by western blotting. Similarly, domain mapping for Hsp40 was carried out using *E.coli* cell lysate expressing GST-tagged full length Hsp40 or J domain or C domain.

### Cell viability assay

Cell viability was assessed using colorimetric based MTT assay. Mock infected or virus-infected A549 cells (MOI = 1) plated into 96-well plates were treated with DMSO or KNK437 for 24 h. 10 μl MTT (Biotium, CA, US) was added to 100 μl of medium in each well and cells were incubated at 37 °C for 4 h. To this, 200 μl DMSO was added and absorbance was measured on spectrophotometer at 570 nm. Background absorbance measured at 630 nm was subtracted from signal absorbance to obtain the normalized absorbance values.

### Mini genome assay

A plasmid based mini genome assay was performed. A549 cells were co-transfected with plasmids for expression of viral polymerase complex proteins (PA, PB1, PB2 and NP) and Renilla luciferase reporter plasmid encoding viral NS1 along with pGL3, which expresses Firefly luciferase and served as an internal control^51^. At 24 h post-transfection, luciferase activity was measured using Dual-luciferase Reporter assay system (Promega, WI, USA). The Renilla luciferase activity was normalized with Firefly luciferase activity and plotted.

### Statistical analysis

Data are expressed as Mean + SD from one representative experiment (n = 3) of at least three independent experiments and comparisons were statistically evaluated by calculating p value using two-tail Student’s t test. P value < 0.05 was considered to be statistically significant.

## Additional Information

**How to cite this article**: Batra, J. *et al.* Human Heat shock protein 40 (Hsp40/DnaJB1) promotes influenza A virus replication by assisting nuclear import of viral ribonucleoproteins. *Sci. Rep.* **6**, 19063; doi: 10.1038/srep19063 (2016).

## Supplementary Material

Supplementary Information

## Figures and Tables

**Table 1:** Sequences of primers used for Real time RT-PCR.

Gene	Primer sequence
β-Actin forward	5′-ACCAACTGGGACGACATGGAGAAA-3′
β-Actin reverse	5′-TAGCACAGCCTGGATAGCAACGTA-3′
NP PR8 forward	5′-CTCGTCGCTTATGACAAAGAAG-3′
NP PR8 reverse	5′-AGATCATCATGTGAGTCAGAC-3′
NP PR8 vRNA	5′-CTCGTCGCTTATGACAAAGAAG-3′
vRNAtag_WSN (for RT)	5′-GGCCGTCATGGTGGCGAAT GAATGGACGGAGAACAAGGATTGC-3′
vRNA tag	5′-GGCCGTCATGGTGGCGAAT-3′
NP WSN reverse	5′-CTCAATATGAGTGCAGACCGTGCT-3′
mRNAtag WSN (for RT)	5′-CCAGATCGTTCGAGTCGT TTTTTTTTTTTTTTTTCTTTAATTGTC-3′
mRNA tag	5′-CCAGATCGTTCGAGTCGT-3′
NP WSN forward	5′-CGATCGTGCCCTCCTTTG-3′
