# Supplementary material for: Human Heat shock protein 40 (Hsp40/DnaJB1) promotes influenza A virus replication by assisting nuclear import of viral ribonucleoproteins
Source: Sci Rep. 2016 Jan 11;6:19063. doi: 10.1038/srep19063 (PMC4707480; doi:10.1038/srep19063)
Supplement: Supplementary Information [file srep19063-s1.pdf]

Human Heat shock protein 40 (Hsp40/DnaJB1) promotes influenza A virus replication by assisting nuclear import of viral ribonucleoproteins

Jyoti Batra[1,2†], Shashank Tripathi[2#†], Amrita Kumar[3], Jacqueline M. Katz[3], Nancy J. Cox[3], Renu B. Lal[3], Suryaprakash Sambhara[3] and Sunil K. Lal[1,2*]

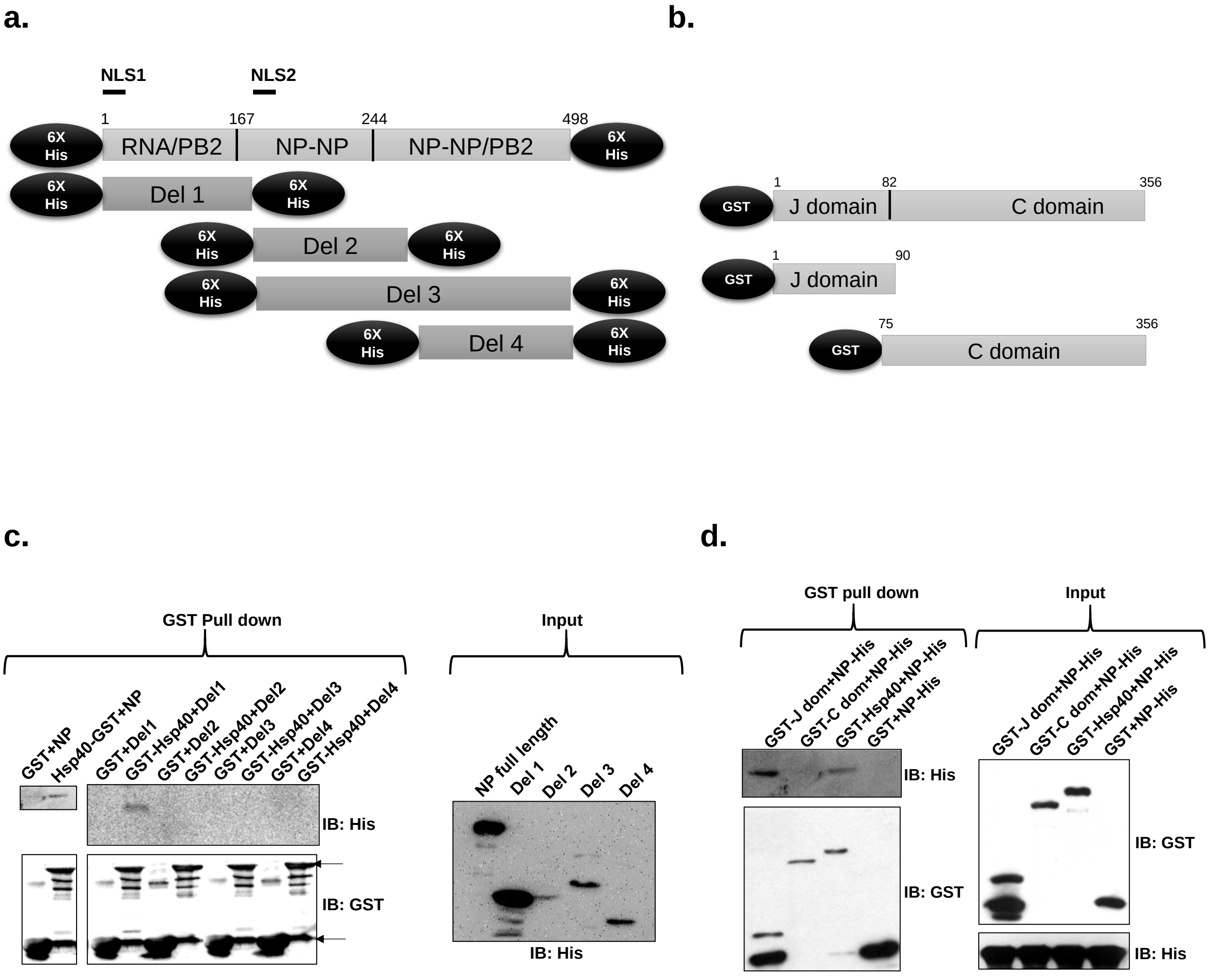

a.
NLS1
NLS2
1
167
244
498
6X His
RNA/PB2
NP-NP
NP-NP/PB2
Del 1
Del 2
Del 3
Del 4
b.
GST
1
82
356
J domain
C domain
90
75
c.
GST Pull down
Input
GST+NP
Hsp40-GST+NP
GST+Del1
GST-Hsp40+Del1
GST+Del2
GST-Hsp40+Del2
GST+Del3
GST-Hsp40+Del3
GST+Del4
GST-Hsp40+Del4
IB: His
IB: GST
NP full length
Del 1
Del 2
Del 3
Del 4
d.
GST pull down
GST-J dom+NP-His
GST-C dom+NP-His
GST-Hsp40+NP-His
GST+NP-His

e.

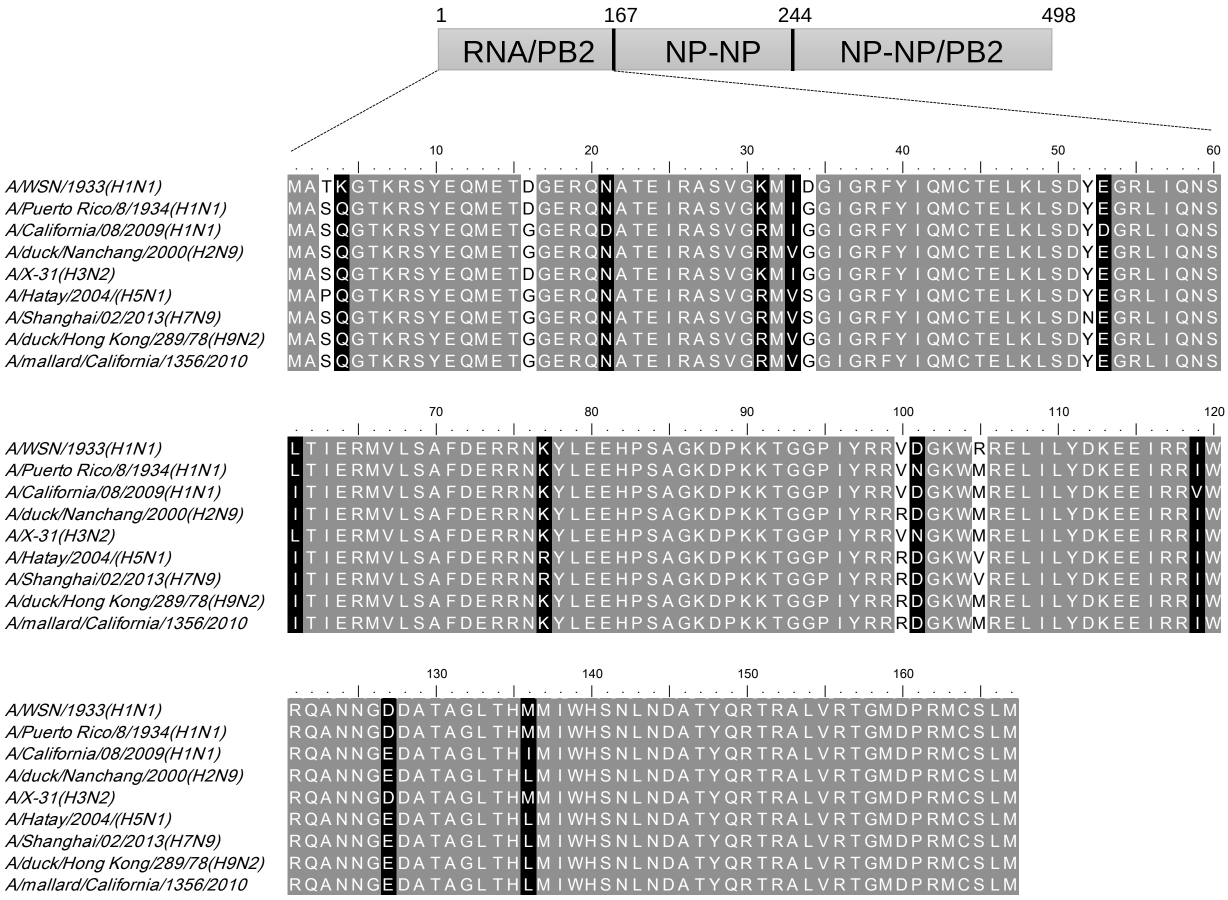

Supplementary Figure S1. NP-Hsp40 interaction is mediated by N-terminal regions of both proteins. (a) Schematics of the IAV NP deletion constructs tagged with N-terminal and C-terminal 6X-His tag. NP functional domains consist of an RNA/PB2 binding domain and two oligomerization domains: NP-1 and NP-2. NLS1 and NLS2 represents location of the nuclear localization sequences. **(b)** Schematic representation of Hsp40 deletions tagged with N-terminal GST tag. **(c, d)** GST pull down assay was performed using bacterially expressed GST-Hsp40 and His tagged full length NP and various NP deletions or GST tagged full length Hsp40 and various Hsp40 deletions and His tagged full length NP. The left panel shows the western blotting of pull down fractions with anti-His and anti-GST antibody to detect NP or NP deletions and Hsp40 or

Hsp40 deletions respectively. The right panel shows the 10% input used for performing GST pull down. Arrows indicate the proteins of interest. **(e)** The N-terminal domain of NP is highly conserved among different subtypes of influenza A virus. Multiple sequence alignment of N-terminal RNA/PB2 binding domain was performed by ClustalW and edited using BioEdit. Identical amino acid residues are represented in grey background and similar residues in black background.

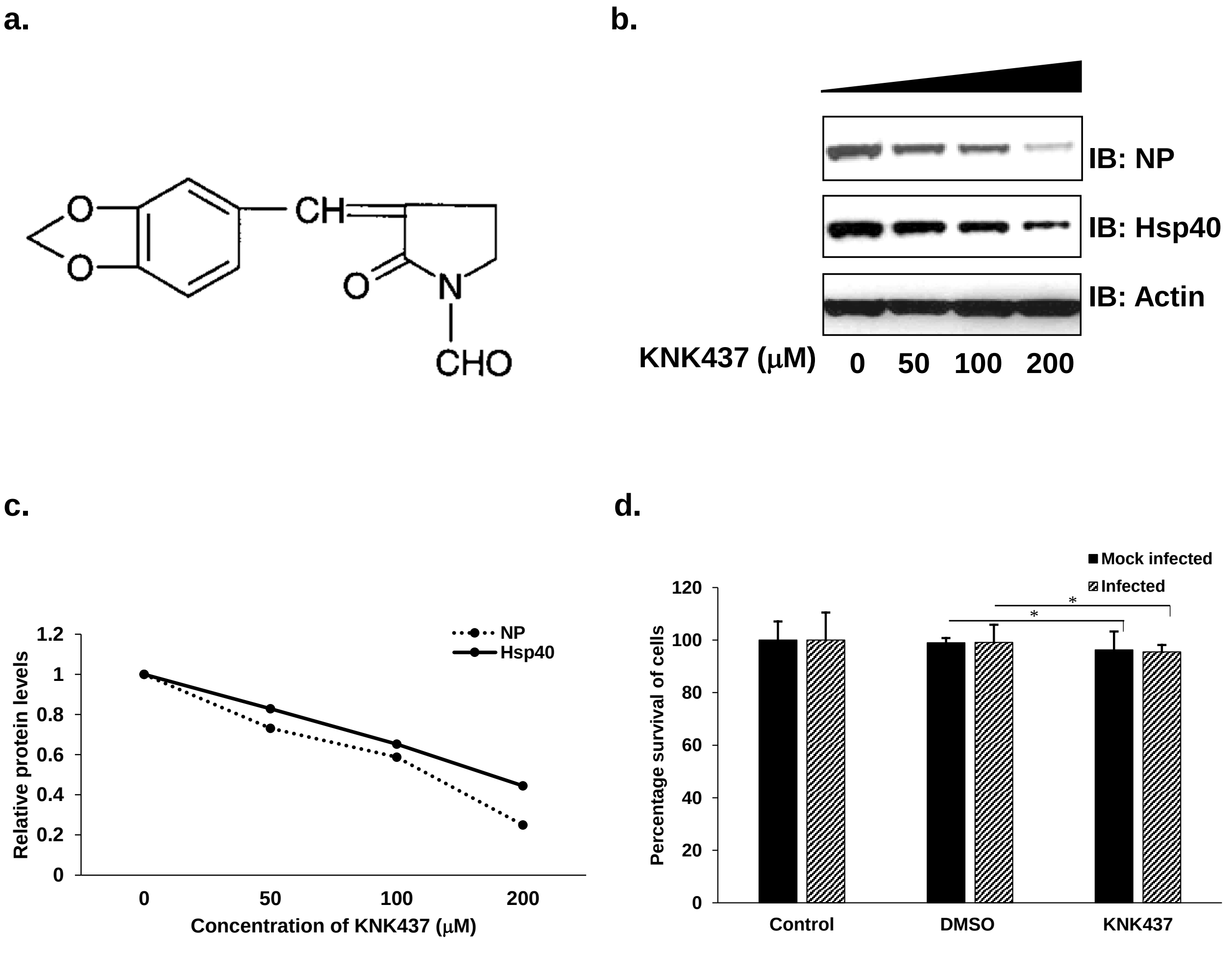

Supplementary Figure S2. KNK437 is a non-cytotoxic inhibitor and inhibits NP levels in dose-dependent manner. (a) The structure of KNK437 (N-formyl-3,4-methylenedioxy-

benzylidene-g-butyrolactam). **(b, c)** Hsp inhibitor inhibits NP levels and corresponding Hsp40 levels in a dose dependent manner. A549 cells were pretreated with DMSO and KNK437 for 6 h and then infected with PR8 virus at MOI=1. 24 h post-infection cells were harvested and expression levels of NP and Hsp40 were analyzed by immunoblotting. Actin was used as a loading control. Densitometry analysis was done using ImageJ and the relative protein levels in KNK437 treated cells to untreated cells were calculated and plotted. **(d)** The cell viability of mock infected cells or cells infected with PR8 virus at MOI=1 and treated with DMSO or KNK437 for 24 h was estimated using MTT assay. Data show mean ± S.D. from one representative experiment (n=3) of at least three independent experiments. An asterisk indicates p>0.05 as calculated by Student's t-test.

a.

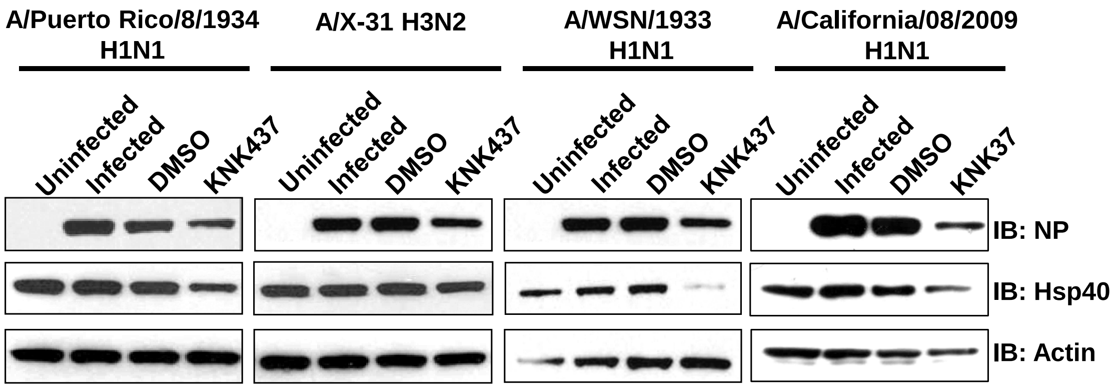

b.

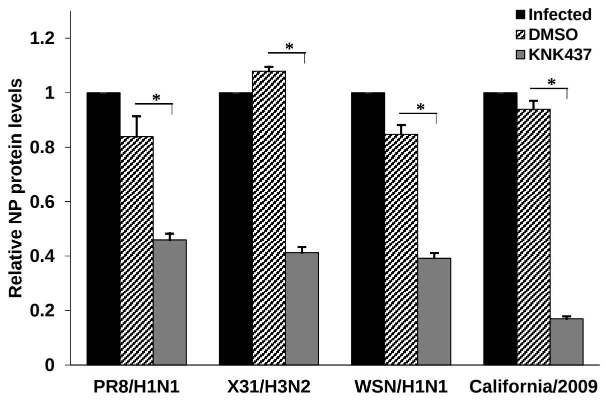

c.

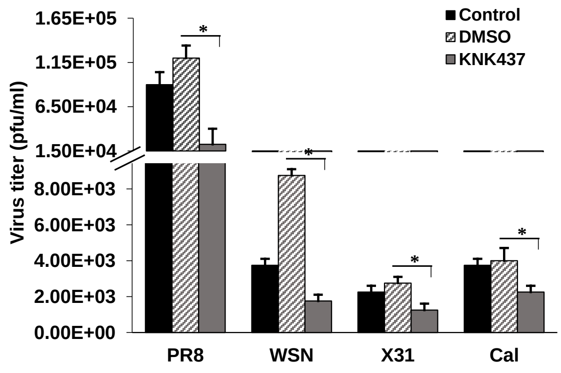

Supplementary Figure S3. KNK437 has inhibitory effect on replication of different IAV isolates. (a, b, c) A549 cells were pretreated with DMSO and KNK437 for 6 h and then infected with different IAV strains at MOI=1. 24 h post-infection, cells were harvested and expression levels of NP and Hsp40 were analyzed by western blotting. Actin was used as a loading control (a). Relative NP protein levels normalized against actin were measured by densitometry and plotted on the graphs (b). Virus titers in the culture supernatants were measured from the same experiment by plaque assay and plotted on the graph (c). Data show mean ± S.D. from one representative experiment (n=3) of at least three independent experiments. Statistical significance was determined using Student's t test. *, $p<0.05$.

a.

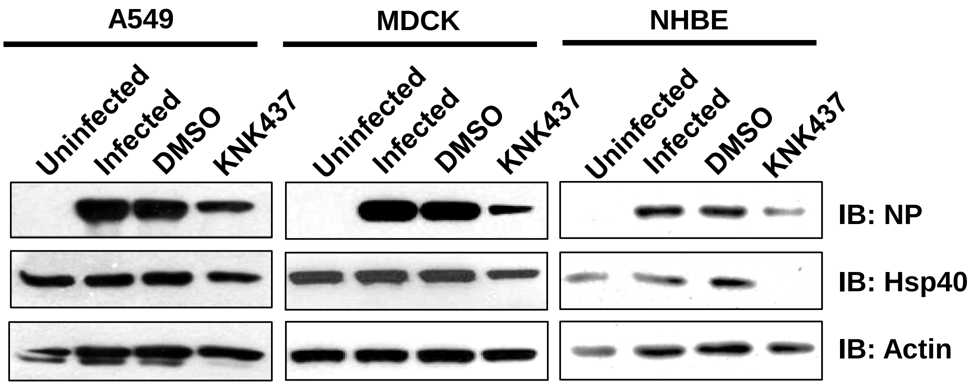

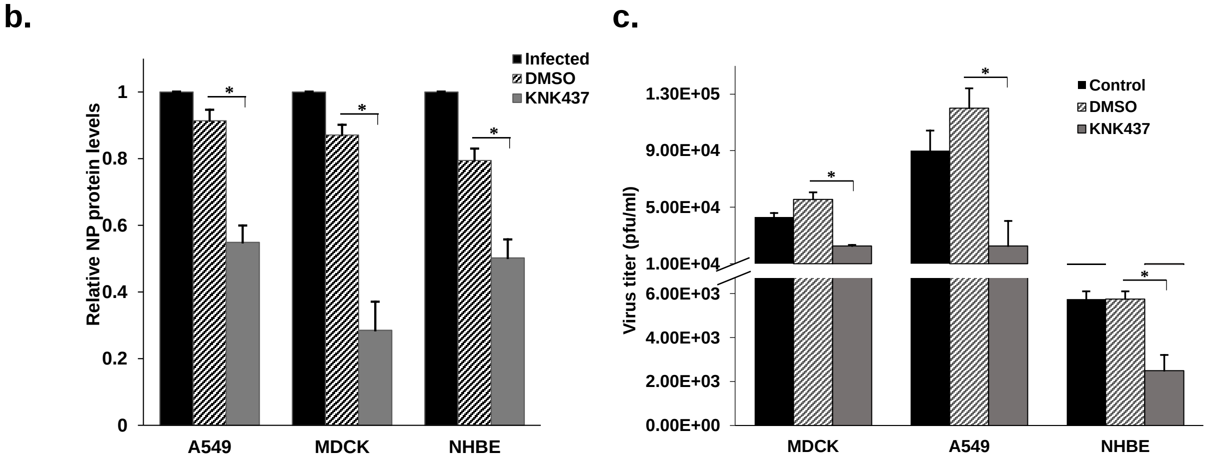

Supplementary Figure S4. Inhibitory effect of KNK437 on IAV replication in different cell lines. (a, b, c) A549, MDCK or NHBE cells were pretreated with DMSO and KNK437 for 6 h and then infected with PR8 IAV at MOI=1. 24 h post-infection, cells were harvested and expression levels of NP and Hsp40 were analyzed by western blotting. Actin was used as a loading control (a). Densitometry analysis was done by ImageJ and relative NP protein levels normalized against actin were plotted (b). Virus titers in the culture supernatants were measured using plaque assay (c). Data show mean ± S.D. from one representative experiment (n=3) of at least three independent experiments. Statistical significance was determined using Student's t test. *, $p<0.05$.

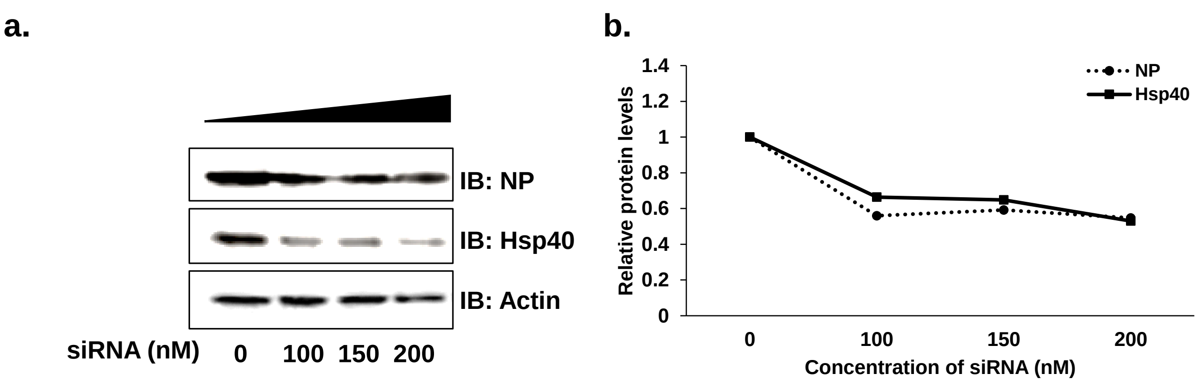

Supplementary Figure S5. Efficacy of siRNA against Hsp40/DnaJB1 (a) Efficacy of siRNA was checked by treating cells with different siRNA concentration for 24 h followed by virus infection at MOI=1. Cells were harvested 24 h post-infection and levels of NP and Hsp40 were analyzed using specific antibodies. **(b)** Densitometry analysis was done using ImageJ. The relative protein levels in siRNA treated cells compared to untreated cells were calculated and plotted.
